# Eccrine poroma presented as spindle-shaped plaque: A case report

**DOI:** 10.1097/MD.0000000000025971

**Published:** 2021-05-21

**Authors:** Yuqian Wang, Meng Liu, Yan Zheng, Yiguo Feng

**Affiliations:** Department of Dermatology, the Second Affiliated Hospital of Xi’an Jiaotong University, School of Medicine, Xi’an, China.

**Keywords:** adnexal neoplasm, eccrine poroma, spindle-shaped plaque

## Abstract

**Rationale:**

Eccrine poroma, a benign cutaneous neoplasm originating from the intraepidermal portion of the eccrine sweat duct, is relatively common in clinical practice. Nevertheless, the 1 presenting as spindle-shaped plaque is extremely rare and easily misdiagnosed as seborrheic keratosis or other dermatoses. Thus, the current study demonstrates a case of eccrine poroma with unique clinical manifestation.

**Patients concerns:**

A 47-year-old man presented with a spindle-shaped plaque on his left sole for 6 years.

**Diagnoses:**

Based on the clinical and histopathological manifestations, diagnosis of eccrine poroma was established.

**Interventions:**

Surgical excision under local anesthesia was performed.

**Outcomes:**

No recurrence or malignant transformation occurred within 6-month follow-up.

**Lessons:**

Eccrine poroma typically presents as a dome-shaped nodule on palm or sole. But this case reminded us the lesion presenting as a spindle-shaped plaque on sole can not rule out the possibility of eccrine poroma.

## 1 Introduction

Eccrine poroma is a benign cutaneous neoplasm originating from the acrosyringium, the intraepidermal portion of the eccrine gland duct. Clinically, poroma typically presents as a solitary, dome-shaped, flesh-colored, dark brown or burgundy papule or nodule which can be either sessile or pedunculated.^[1,2]^ It is generally slowly growing. Diameter of the protruding lesion ranges from a few millimeters to 2 centimeters and the surface is usually smooth or slightly lobulated.^[3,4]^ It can be located on almost any cutaneous surface while sole and palm are most common locations.^[1,5]^ To the best of our knowledge, few cases of plaque-like eccrine poroma have been reported. In order to raise awareness among dermatologists with regard to the rare behavior of eccrine poroma, we demonstrate a unique poroma case presenting as a spindle-shaped plaque in a male patient.

## 2 Case report

**Figure 1:**
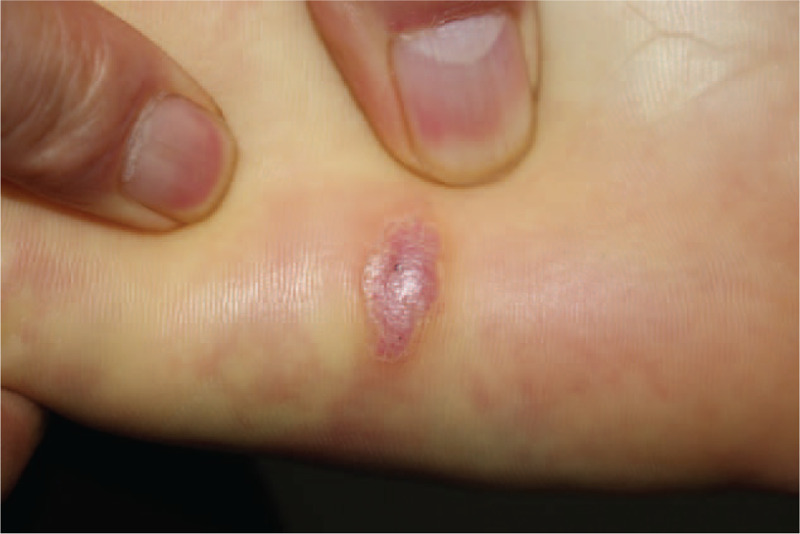
The solitary, reddish, elevated, spindle-shaped plaque with tenderness on the left sole.

A 47-year-old man presented with a plaque (1.0 cm in length, 0.5 cm in width) on his left sole (Fig. 1). The lesion started 6 years before without history of trauma or chronic irritation at the site of the tumor. As it was slowly enlarging, he began to feel tenderness, with no itching, bleeding or other discomforts. There is no similar lesion or other tumor history in his family members. The patient was generally in good condition. No changes in diet, sleep, urination, defecation, or body weight were reported. Physical examination revealed stable vital signs. The systemic superficial lymph nodes, liver, and spleen were not involved, and the systematic examination showed no evident abnormalities. A dermatological examination revealed a well-defined, reddish, protruding, spindle-shaped plaque on his left sole with rough surface, tough texture, poor activity, and a little tenderness. The tumor was not adherent to subcutaneous tissues or other underlying structures and no inflammatory changes were noted.

**Figure 2:**
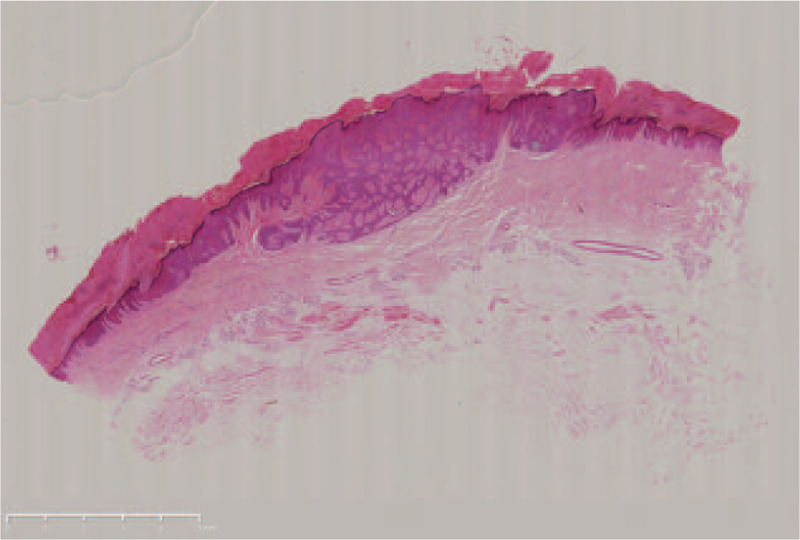
Hematoxylin-eosin (H&E) staining of the lesion, showing broad anastomosing bands extending from the basal epidermis deep into the dermis. Bar length = 10 mm.

**Figure 3:**
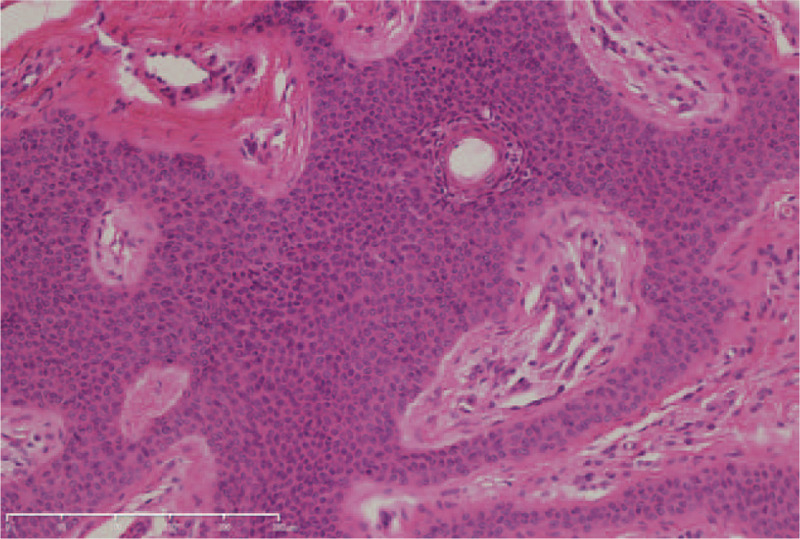
The tumor cells were monomorphous and cuboidal and had deep basophilic round nuclei (H&E). Bar length = 250 μm.

There were no obvious abnormalities in blood routine test, coagulation and liver and kidney function. Under local anesthesia the lesion was completely resected with a 2-mm margin and histopathological evaluation was performed. Histopathology findings revealed broad anastomosing bands comprising of poroid cells extending from the basal epidermis deep into the dermis (Fig. 2). The tumor cells which were smaller than keratinocytes were monomorphous and cuboidal and had deep basophilic round nuclei (Fig. 3). Based on the clinical and histopathological manifestations, diagnosis of eccrine poroma was established. No recurrence or malignant transformation occurred within a 6-month follow-up.

## 3 Discussion

Eccrine poroma, which was first reported by Goldman et al in 1956, is a rare adnexal neoplasm originating from the acrosyringium, the intraepidermal portion of the eccrine sweat duct, representing about 10% of sweat gland tumors, and less than 1% of all primary dermatosis.^[2,4]^ Etiology of the disease has not been elucidated but it is speculated to have association with radiation, trauma, pregnancy, chemotherapy drugs and infection of human papillomavirus.^[1,2,5]^ Our patient had no predisposing or associated factors, which indicated further study is needed to clarify mechanisms facilitating poroma. Recently, a study on molecular basis of eccrine poroma has reported that 88.5% of poroma patients present with recurrent yes-associated protein-mastermind-like protein 2 and yes-associated protein-NUT family member 1 fusion and a WWTRQ-NUT family member 1 fusion was encountered in a single lesion of poroma. The fusions strongly transactivated a TEA domain receptor and caused abnormal cell proliferation, thereby resulting in eccrine poroma.^[6]^

Clinically, poroma tends to occur in middle-aged people to the elderly and its most common locations are sites with a large aggregation of eccrine sweat glands like soles and palms while other sites such as scalp, eyelid, nose, neck, trunk and lower extremity were also reported.^[1,5,7]^ The lesion typically presents as a solitary, exophytic, dome-shaped, flesh-colored, dark brown or burgundy papule or nodule which can be either sessile or pedunculated.^[1,2]^ Diameter of the protruding lesion ranges from a few millimeters to 2 centimeters and the surface is usually smooth or slightly lobulated.^[3,8,9]^ It is generally slowly growing. Only 2 cases which reported eccrine poromas presenting as plaque were retrieved from PubMed, of which one was diagnosed as collision tumor of eccrine poroma, seborrheic keratosis, and a viral wart, and another presented as a sessile pink plaque with a coalescing papular texture.^[10,11]^ In our patient, a spindle-shaped plaque can be observed, which was different from previously reported cases with papules or nodules and reminded us the lesion presenting as a plaque on sole or palm can not rule out the possibility of eccrine poroma. Of note, sudden pruritus, pain, bleeding and ulcers following irritation or trauma may indicate the mass has progressed to eccrine porocarcinoma.^[12]^

The diagnosis of eccrine poroma mostly relies on the histopathological examination. Tumor histology reveals that the broad anastomosing bands comprising of poroid cells extend from the epidermis downward into the dermal layer. The tumor masses consist of monomorphous, cuboidal and deep basophilic cells which are smaller than adjacent normal keratinocytes. Round or ovoid nuclei with clear cytoplasm and focal ductal differentiation can be seen in microscopic examination while no peripheral palisade presents.^[1,2]^ Furthermore, there might be mitoses, but no cellular atypia can be found.^[13]^ The poroma is characterized by epithelial membrane antigen and carcinoembryonic antigen positive and Ber-Ep4 negative.^[2]^

Clinically, eccrine poroma is easily misdiagnosed as verruca, pyogenic granuloma and seborrheic keratosis. Thus, the histopathological examination is the golden standard for a definite diagnosis: Verruca is characterized by epidermal hyperplasia, hyperkeratosis, koilocytosis in granular layer. Histopathological findings of pyogenic granuloma highlight significant amounts of capillaries embedded in an edematous stroma and infiltration of polymorphonuclear leukocytes with granulation tissue. As for seborrheic keratosis, which typically presents as a brown/brown-blackish, slightly elevated maculopapule, needs differential diagnosis with plaque-like eccrine poroma just like our case due to the mimetic appearance. Histopathological analysis of seborrheic keratosis reveals that the tumor masses compose of basaloid cells, hyperkeratosis and horn cysts can be seen which are totally distinct from poroma. ^[14,15]^

Histopathologically, squamous cell carcinoma and superficial basal cell carcinoma must be ruled out.^[16]^ Diagnosis of squamous cell carcinoma is based on squamous cells masses extending into the dermis, with keratinizing tendency. Squamous peals and dyskeratotic cells can be seen in the lesion.^[17]^ Superficial basal cell carcinoma, the tumor cells of which are mainly basaloid cells, is characterized by peripheral palisade and the clefting between the tumor and adjacent tissues. In addition, basal cell carcinoma is Ber-Ep4 positive which is inconsistent with poroma.^[2]^

There are various nonsurgical treatment including the laser, electrocautery and liquid nitrogen cryotherapy, but surgical excision is the optimal choice of eccrine poroma.^[2,5,18]^ It is worth noting that a regular follow-up is emphasized after excision due to the possibility of malignant transformation and recurrence.^[2]^

In conclusion, the present case manifested with plaque-like appearance, instead of the commonly observed nodules. To the best of our knowledge, eccrine poroma with such manifestation has rarely been reported. We demonstrate this unique poroma case in order to raise awareness among dermatologists with regard to the rare behavior of eccrine poroma and decrease the misdiagnosis rate.

## Author contributions

**Conceptualization:** Yan Zheng, Yiguo Feng.

**Methodology:** Yiguo Feng.

**Project administration:** Yuqian Wang.

**Supervision:** Yan Zheng, Yiguo Feng.

**Writing – original draft:** Yuqian Wang, Yiguo Feng.

**Writing – review & editing:** Yuqian Wang, Meng Liu, Yan Zheng, Yiguo Feng.
